# Strain-specific nuclear genetic background differentially affects mitochondria-related phenotypes in *Saccharomyces cerevisiae*

**DOI:** 10.1002/mbo3.167

**Published:** 2014-04-02

**Authors:** Arianna Montanari, Silvia Francisci, Mario Fazzi D'Orsi, Michele Maria Bianchi

**Affiliations:** 1 Department of Biology and Biotechnologies “C. Darwin”, Sapienza University of RomePiazzale A. Moro 5, Rome, 00185, Italy; 2 Pasteur Institute-Cenci Bolognetti Foundation, Sapienza University of RomePiazzale A. Moro 5, Rome, 00185, Italy

**Keywords:** Diseases, nuclear background, oxidative stress, respiration, yeast model

## Abstract

In the course of our studies on mitochondrial defects, we have observed important phenotypic variations in *Saccharomyces cerevisiae* strains suggesting that a better characterization of the genetic variability will be essential to define the relationship between the mitochondrial efficiency and the presence of different nuclear backgrounds. In this manuscript, we have extended the study of such relations by comparing phenotypic assays related to mitochondrial functions of three wild-type laboratory strains. In addition to the phenotypic variability among the wild-type strains, important differences have been observed among strains bearing identical mitochondrial tRNA mutations that could be related only to the different nuclear background of the cells. Results showed that strains exhibited an intrinsic variability in the severity of the effects of the mitochondrial mutations and that specific strains might be used preferentially to evaluate the phenotypic effect of mitochondrial mutations on carbon metabolism, stress responses, and mitochondrial DNA stability. In particular, while W303-1B and MCC123 strains should be used to study the effect of severe mitochondrial tRNA mutations, D273-10B/A1 strain is rather suitable for studying the effects of milder mutations.

## Introduction

*Saccharomyces cerevisiae* has been extensively used to understand the genetic and molecular aspects of cellular roles of mitochondria within the cells and to study the regulation of mitochondrial (mt) functions, due to its exceptional capability to grow by fermentation when respiration is absent, repressed, or severely impaired. In glucose-containing media, respiration-deficient mutants can be easily identified because they grow slowly, forming small (*petite*) colonies that are OXPHOS-deficient mutants carrying partial deletions of mt DNA (rho^−^ *petites*) or mt DNA-less strains (rho° *petites*).

*Saccharomyces cerevisiae* has been proposed as a model for the study of several mt pathologies (Koutnikova et al. 1997; Facchin et al. 2003; Feuermann et al. 2003; Valente et al. 2007; Montanari et al. 2010; Doimo et al. 2012; Panizza et al. 2013) as it is able to survive without functional mitochondria. Mitochondrial pathologies can be due to mutations in nuclear or mt genes affecting mt processes such as protein synthesis, assembly of respiratory complexes, synthesis of Fe-S centers, mt import/export; moreover several other serious diseases, including Parkinson and Huntington diseases, certainly have a mt link.

Mt gene expression requires the coordinate participation of nuclear and mt products. The regulatory interactions between these components, and the *cis*-acting signals necessary for the control of the mt transcription and translation are still only partially understood.

In the last 60 years, the selection and genetic characterization of laboratory strains of *S. cerevisiae* went on, starting from the previously isolated beer or baker's strains, to obtain haploid heterothallic strains in which genetic analysis was possible (Lindegren and Lindegren 1971). The characters selected were initially the capability of sugar fermentation, the easiness to isolate nutritional mutants, and the absence of clumpyness (necessary to isolate individual colonies). Bob Mortimer isolated the strain S288C (Mortimer and Johnston 1986), which is the main source of the strains present in most laboratories and from this extensively studied strain, the BY 4743 and W303-1B, used for genome sequencing (see http://www.yeastgenome.org), were derived. A different strain, D273-10B/A1, has been widely used to study the mitochondrial complexes (Sherman et al. 1968; Tzagoloff et al. 1975) and certainly has a different origin as its mt genome is smaller compared to the previously mentioned strains, due to the absence of several introns and to differences in the number of G+C clusters. In the present report, we examined several aspects of mt functions of the two representative strains D273-10B/A1 and W303-1B, and of strain MCC123, which has been selected by T. Fox for mt biolistic transformation (Mulero and Fox 1993).

In the course of our studies on mt tRNA mutants (De Luca et al. 2009; Montanari et al. 2010, 2011), we have previously observed important phenotypic differences among *S. cerevisiae* strains, indicating that a better definition of the involved genetic variability will be essential to define the relationship between the mt efficiency and the characteristics of different nuclear backgrounds.

In order to compare the phenotypic effect of the same mt mutation in the presence of different nuclear backgrounds, mitochondria containing a mutated tRNA gene were transferred by cytoduction crosses from the MCC123 strain into D273-10B/A1 and W303-1B rho° cells. Here, we demonstrated that phenotypes of the yeast mt tRNA mutants were strongly dependent on the nuclear background. Although it is not yet clear what accounts for the strains differences, it is likely that parallel retrograde signals (from mitochondria to nucleus) or anterograde signals (from nucleus to mitochondria) operate in response to mt perturbations with different targets and effectors. Results showed that specific strains might be used preferentially to evaluate specific mt functions and the effect of different mt mutations.

## Materials and Methods

### Yeast strains and growth conditions

*Saccharomyces cerevisiae* WT strains used were the MCC123 (*MATa, ade2, ura3-52, Δleu2::KanR, kar1-1, rho*^*+*^; Mulero and Fox 1993; the W303–1B (*MATα, ade2-1, can1-100, his3-11,15, leu2-3,112, trp1-1, ura3-1*; Thomas and Rothstein 1989), the D273–10B/A1 (*MATα, met6, ura3-52*; Sherman and Slonimski 1964), and the FF1210-6C (*MATα, ura1, ura2*; Elelj-Fridhi et al. 1991). The latter strain has been used to generate the biolistic mt tRNA-mutant strains (YGM strains), obtained as previously described in Feuermann et al. (2003). By cytoduction crosses, we transferred WT and mutated mitochondria from FF1210-6C into the rho° cells of the three WT strains; the procedure is described in detail in De Luca et al. (2009). The mutant strains were named with the first letter of the isogenic WT and a three-letter code name of the amino acid indicating the mutated mt tRNA gene and the position of the base substitution referring to yeast cytoplasmic tRNA^Phe^ (Sprinzl and Vassilenko 2005).

Strains were grown in YP complete medium (1% yeast extract and 1% peptone from Difco) containing 3% glycerol or glucose (2% or 0.25%). Comparison of growth capability was investigated by serial dilutions of concentrated suspensions (5 × 10^6^ cell/mL), prepared from fresh single colonies, spotted onto a unique plate. Pictures were acquired after 2–3 days of growth on glucose plates and after 5 days of growth on glycerol plates.

To measure the production of rho^−^/rho° colonies, a large fresh colony grown on YP-2% glucose plate was inoculated in the same liquid medium. After an overnight growth, the culture was diluted and plated; after 2–3 days of growth and large and small colonies were counted.

### Stress resistance assays

Sensitivity to EGTA was performed by spotting serial dilution of exponentially growing cells on YP-2% glucose medium containing 20 or 30 mM EGTA.

Sensitivity to hydrogen peroxide was assayed by halo assay performed by plating exponentially growing cells (approximately 2 × 10^7^ cells/mL in YP-2% glucose liquid medium) at high density on YP-2% glucose; a filter disk saturated 50 mM H_2_O_2_ was then placed on the medium surface**.** After an overnight incubation at 28°C, the halo sizes of growth inhibition were measured.

For chronological lifespan determination, cells were inoculated in YP-2% glucose liquid medium. As the culture reached the stationary phase, the same volumes of 10^−5^ dilutions were plated every 2–3 days. Colonies were counted after 4-days incubation at 28°C. In the same plates, we discriminated the *petite* colonies formation from the rho^+^ and we calculated the rho°/rho^−^ percentages. Data were mean values of three independent experiments and standard deviations were calculated. Statistical analysis was performed with the Student's *t*-test.

### Microscopy

Cells from exponential cultures were stained with 1 *μ*M DASPMI (2-(4 dimethylaminostyryl)-*N*-methylpyridinium iodide), as described by Rafael and Nicholls (1984), and were observed immediately by fluorescence microscopy.

### Cultivation in bioreactor and respiration studies

We used a BiostatQ (B-Braun, Melsungen, Germany) bioreactor endowed with four 1-liter vessels, each containing 600 mL of YP 0.25% glucose and inoculated with late log precultures, grown in YP containing 2% glucose, at starting cell concentrations of 0.5–1 × 10^7^ cell/mL. Incubations were performed at 28°C with magnetic stirring (300 rpm) and air supply (0.4 L/min).

Respiration studies were performed using a Clark oxygen electrode (Hansatech Instruments) as previously described (De Luca et al. 2009). The pellet corresponding to 0.03 g (wet weight) was collected from a fresh colony, washed with 1 mL sterile water, suspended in 1 mL of Na-phosphate buffer 10 mM pH7.4 containing 0.4% glucose, and loaded in the Reaction Vessel of the previously calibrated Oxygen Electrode Chamber.

## Results

### Comparison of growth and respiration among WT strains

**Figure 1:**
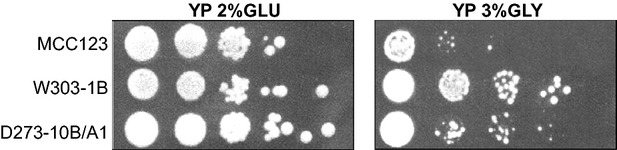
Growth capability of WT strains. Serial dilutions of cultures from three WT strains were spotted on YP plates containing 2% glucose or 3% glycerol as carbon sources and incubated at 28°C. Pictures were acquired after for 2–3 days of growth for plates containing glucose, and after 5 days of growth for plates containing glycerol as carbon source.

We have compared the growth capability of the three WT strains W303-1B, D273-10B/A1, and MCC123. Cells grown overnight in YP-2% glucose liquid medium to exponential phase were spotted by serial dilution on fermentable (glucose) or respirable (glycerol) solid media. Figure 1 shows that although the strains grew similarly on YP-2% glucose-containing plates, on glycerol plates (respiring condition) the W303-1B strain had the best growth while MCC123 showed very low growth, both at 28°C or 37°C (not shown). Strain D273-10B/A1 showed an intermediate behavior.

**Figure 2:**
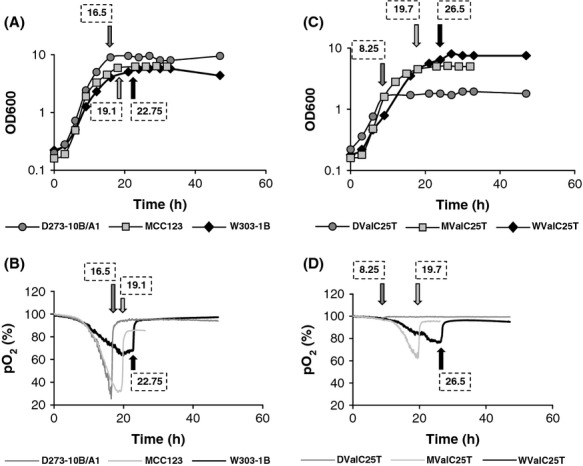
Cultivation in bioreactor of WT cells and mt tRNA-mutant cells. Comparison of growth curves and of O_2_ consumption rates of WT strains (panels A and B) and of WValC25T, MValC25T, and DValC25T mutants (panels C and D) performed in YP 0.25% glucose-containing medium. In panels B and D, the times of respiration arrest are indicated by arrows flagged with time values. The same flagged symbols are reported in panels A and C at the corresponding time points.

**Figure 3:**
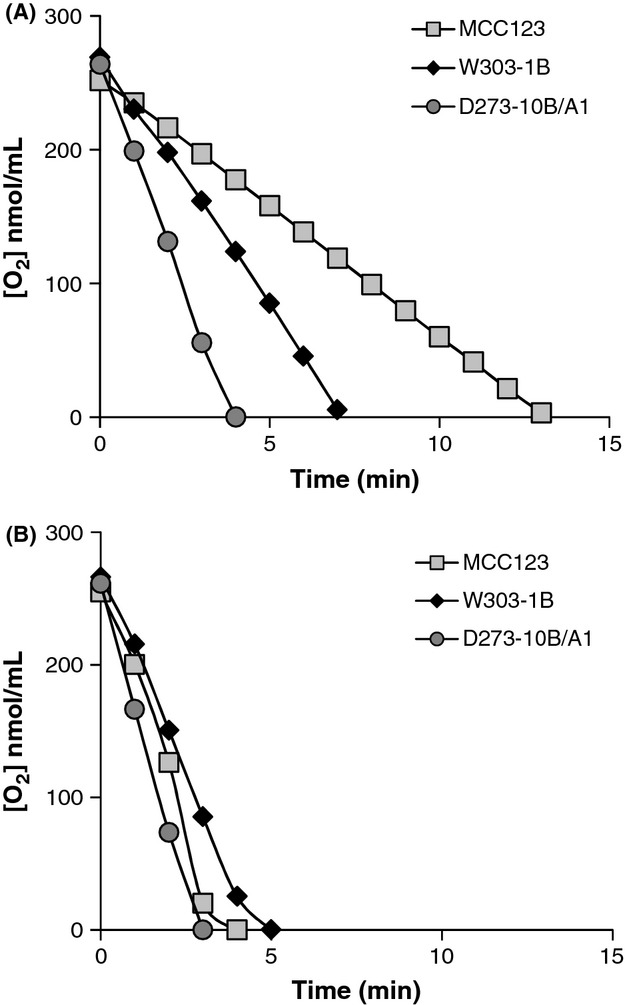
Oxygen consumption curves of resting WT strains. Comparison of Oxygen consumption rates in reaction chamber of WT cells in resting condition. Cells were previously grown in YP medium containing 2% (A) or 0.25% glucose (B).

To verify if the difference in growth capability observed on YP plates containing 3% glycerol as carbon source (Fig. 1) could be ascribed to different cell duplication times due to possible variable respiratory efficiency, we performed the growth curve in bioreactor for the three WT strains, measuring the optical density of the cultures. In parallel, we monitored the dissolved oxygen concentration with in situ electrodes. The experiments were performed in YP medium containing 0.25% glucose. This low glucose concentration was chosen to allow respiratory metabolism by minimizing glucose repression (see Fig. 3). We did not use glycerol that is metabolized only by respiring cells in order to permit the comparison of growth capability with mt-mutant strains unable to grow on this carbon source (see below). Profiles of representative experiments are reported in Fig. 2. Comparison of the growth curves (Fig. 2A) showed a substantial difference in the duplication rate (calculated at the exponential growth phase) for the MCC123 (1.8 h) and the W303-1B (2.8 h) with the D273-10B/A1 showing an intermediate duplication rate (2.1 h). The OD yields at the stationary phase were 9–9.5 for the D273-10B, 6.2 for the MCC123 and 5.5–5.7 for the W303-1B.

Oxygen consumption was faster and continuous in the D273-10B/A1 and MCC123 cultures compared to the W303-1B, where oxygen consumption slowed down at the end of the exponential growth before entering in the stationary phase (Fig. 2B). All together the profiles of Fig. 2 show that D273-10B/A1 culture rapidly reached the stationary phase and abruptly stopped to respire after 16.5 h, whereas the W303-1B strain shifted slowly to stationary phase after 22.75 h. The MCC123 culture showed fast oxygen consumption similar to the D273-10B/A1 but with a difference in the extent of respiration; in fact, the MCC123 cells continued to respire also at the beginning of the stationary phase until 19.1 h.

To better understand the differences in the rate of oxygen consumption, we compared the respiration of resting cells previously grown in YP media containing 0.25% or 2% glucose as carbon sources. In this experiment, oxygen consumption was measured in an Oxygen Reaction Chamber (Fig. 3). The oxygen consumption rates were very different among the WT strains (D273-10B/A1 > W303-1B > MCC123; see Fig. 3A) for cells grown on fermentative/glucose-repression conditions (2% glucose), whereas minor differences were observed when cells were allowed/forced to respire by the low glucose concentration (0.25%; see Fig. 3B). The above results demonstrated a variable glucose-repression sensitivity among the WT strains, while the respiration capability of the three strains was substantially similar.

### Comparison of growth and respiration among mt tRNA mutants

In a previous work (De Luca et al. 2009), we analyzed the phenotype of the mutants bearing an identical mt tRNA substitution (LeuA29G) in different strains and found that the severity of respiratory defects followed this trend: D273-10B/A1 > MCC123 > W303-1B. This behavior should be ascribed only to the influence of the nuclear background as the mutated mitochondria were transferred by cytoduction crosses in the different rho° strains. By following this procedure, both nuclear and mt interstrains recombination were prevented and mt DNA was maintained (De Luca et al. 2009).

In the present work, we extend our investigation to other mt tRNA mutants (LeuT20C and ValC25T) to evaluate how the different nuclear background can influence the respiratory phenotypes. To this purpose, we intentionally excluded mt mutations that completely abolished glycerol growth in each nuclear background and we choose mutants whose mt protein synthesis might be only partially affected. In fact, previous data showed that the LeuT20C mutation produced a conformational change in the three-dimensional structure impairing the tRNA^Leu^ aminoacylation at 37°C (Montanari et al. 2008); a generalized decrease in mt transcription for the LeuA29G mutant was observed; no detectable tRNA^Val^ was reported for ValC25T cells (De Luca et al. 2009). In Fig. 3C, D we show the cultivation in bioreactor of three strains harboring the C25T substitution in the same mt tRNA gene (tRNA valine), performed identically to the WT strains. The mutated mitochondria were introduced by cytoduction (see Materials and Methods) from the MValC25T strain (MCC123 WT parental strain) into W303-1B and D273-10B/A1 rho° cells. The mutants were named by their mutation type and position, according to the standard tRNA numbering (Sprinzl and Vassilenko 2005); the first letter corresponded to their isogenic WT strain.

The effect of the mt mutation on growth in bioreactor was evident only in the D273-10B/A1 nuclear background. In fact, growth of DValC25T in YP-0.25% glucose-containing medium was supported only for the early exponential growth phase when presumably the cells consumed the endogenous pool of metabolites till exhaustion. Duplication was arrested after about 9 h of growth (duplication time was 2.8 h, OD yield 1.8: see Fig. 2C). Differently, the WValC25T and MValC25T mutants grew very similarly to their WT strains, although the WValC25T showed a slightly longer duplication time (3.3 h) and slightly higher biomass yield (7.5 OD), than its corresponding WT strain, reaching the stationary phase after 26.5 h of growth. Oxygen consumption profiles of the three mt tRNA^Val^ mutants overlapped for the first 8 h of growth, but after longer time, substantial differences are shown: in DValC25T oxygen consumption and growth stopped after 8.25 h; in WValC25T growth continued and stopped respiring after 26.5 h, similar to the isogenic WT; the MValC25T exhibited an intermediate behavior with an arrest in respiration after 19.7 h (Fig. 2D). In conclusion, the ValC25T mutation in D273-10B/A1 nuclear background did not permit respiration; in the MCC123 background, the mutation induced a significant decrease in respiration (from 20 to 60% of residual O_2_), while in the W303-1B nuclear background had only a minor effect.

**Figure 4:**
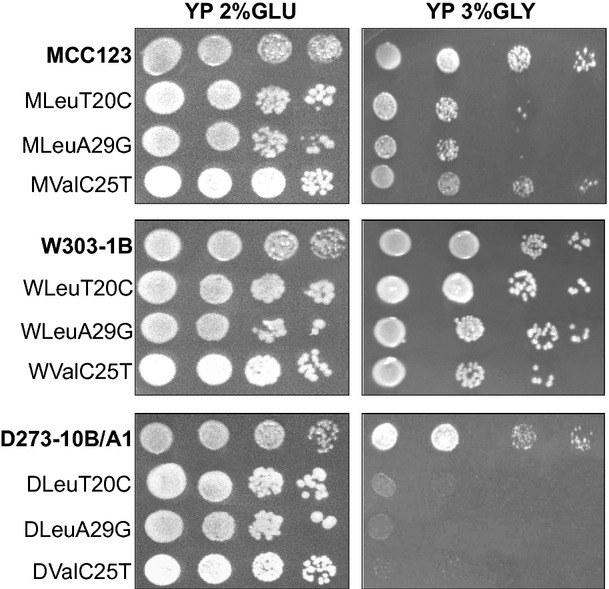
Influence of three different nuclear backgrounds on the glycerol growth phenotype of mt tRNA mutants. Serial dilutions of LeuT20C, LeuA29G and ValC25T yeast mutant strains, having MCC123, W303-1B and D273-10B/A1 as nuclear background, grown overnight in YP liquid medium containing 2% glucose, were spotted on a YP plate containing 2% glucose or 3% glycerol and incubated at 28°C for 7 days.

The comparison of growth capability of mt tRNA mutants was also studied by serial culture dilutions spotted on plates containing 2% or 3% glycerol as carbon sources and incubated at 28 and 37°C. As we obtained similar results at both temperatures, in Fig. 4, we show only plates incubated at 28°C. For each nuclear background, we also analyzed the growth phenotype of two mutations located in the tRNA^Leu^ (LeuT20C, LeuA29G) in addition to the C25T mutation in the tRNA^Val^**.** In YP-2% glucose plates all mutants were found to grow similar to their WT. Differently, no defective growth phenotype was observed for the tested mutants in the MCC123 nuclear background on YP-glycerol medium, whereas, in the D273-10B/A1 background, all the mutations analyzed completely abolished glycerol growth (Fig. 4). A mild defective growth was observed in the W303-1B background only for mutant WValC25T (Fig. 4).

**Figure 5:**
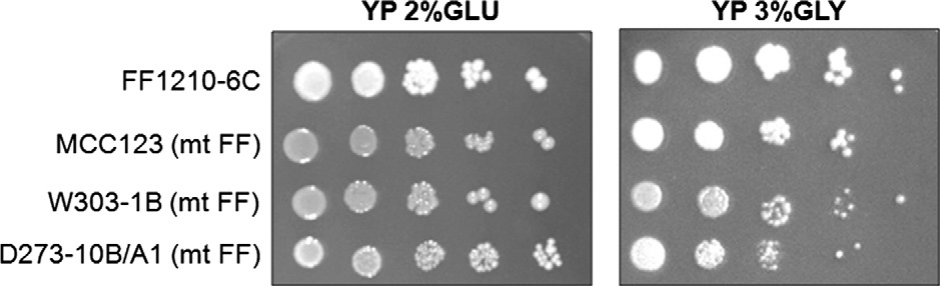
Influence of three different nuclear backgrounds associated to the same WT mt DNA on the glycerol growth phenotype. Serial dilutions of the three strains MCC123, W303-1B and D273-10B/A1, endowed with WT mitochondria of the strain FF1210-6C, were grown overnight in YP liquid medium containing 2% glucose, spotted on a YP plate containing 2% glucose or 3% glycerol and incubated at 28°C for 5 days.

The phenotypic variability observed in Fig. 4 was not due nucleomitochondrial incompatibilities as FF1210-6C WT mitochondria, introduced by cytoduction crosses in rho° cells derived from the three WT strains, allowed similar growth capability in glucose- and in glycerol-containing media (Fig. 5). Strain FF1210-6C was the strain used to generate the mt tRNA-mutant strains here described.

### Rho^−^/rho° production in WT and tRNA mutants

It is known that inhibition of mt protein synthesis increases the formation of petite mutants (Myers et al. 1985); these cells have partially or completely lost their mt DNA. We have previously shown that within a specific nuclear background, it is possible to use the percentage of rho^−^/rho° production as an indicator of the importance of the mt protein synthesis defect (De Luca et al. 2009). We report here (Table 1) that after an overnight growth in YP containing 2% glucose, the three WT strains had similar rho^−^/rho° percentage (2%). Table 1 also shows that this value was maintained in the analyzed mt tRNA-mutant strains having the D273-10B/A1 nuclear background, whereas when the mutated mitochondria were transferred to the other nuclear backgrounds by cytoduction procedure, a statistically significant increase in rho^−^/rho° production was observed. In particular, in MCC123 and in W303-1B cells, the mutated mt DNA was very unstable depending on the mt tRNA substitution. This behavior has been observed for other mt tRNA mutations and we have previously shown that the rho° production could reach 80% for the human equivalent MELAS substitutions (Feuermann et al. 2003; Montanari et al. 2008).

**Table 1:** Generation of *petites* in wild type and mt tRNA mutant strains

Strain	rho^−/^° percentage, %	References
MCC123	2	Montanari et al. (2008)
MLeuT20C	35	Montanari et al. (2008)
MLeuA29G	45	De Luca et al. (2009)
MValC25T	27	De Luca et al. (2009)
W303-1B	2	Montanari et al. (2008)
WLeuT20C	13	Montanari et al. (2008)
WLeuA29G	11	This paper
WValC25T	22	This paper
D273-10B/A1	2	Montanari et al. (2008)
DLeuT20C	2	Montanari et al. (2008)
DLeuA29G	2	This paper
DValC25T	2	This paper

### Stress resistance assays comparison

We tested how mt tRNA-mutant cells responded to stress assays and if protection mechanisms were activated similarly in different nuclear contexts. For this purpose, a halo assay sensitive to hydrogen peroxide with cells having different respiratory ability (WT, isogenic rho° petite and mt tRNA-mutant cells) was performed.

**Figure 6:**
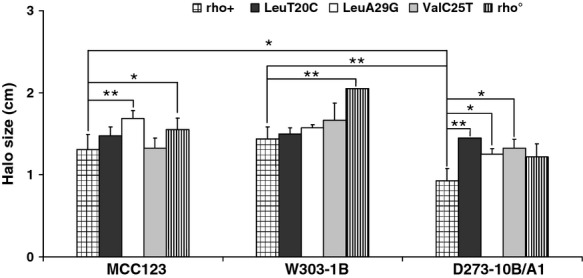
Hydrogen peroxide sensitivity of WT, rho° and mt tRNA mutants in MCC123, W303-1B, and D273-10B/A1 nuclear backgrounds. Comparison of the halo sizes of H_2_O_2_ growth inhibition (disks saturated with 50 mM H_2_O_2_) obtained by plating 2 × 10^9^ cells from exponential cultures. The values of halo sizes, expressed in cm as halo radius after subtraction of disk radius, are means (with standard deviations) obtained from three independent experiments. *(p < 0.05) and **(p < 0.01) indicate the statistical significance of differences calculated by comparing the strains linked by the bars.

The results showed that strains having defective respiration (rho° cells and mt tRNA mutants) were in general all more sensitive to H_2_O_2_ compared to the isogenic WT (Fig. 6). However, in the W303-1B background, where the presence of rho° mitochondria produced the higher increase in sensitivity, the mt tRNA mutations did not cause a significant variation in sensitivity compared to the isogenic WT. Only in the MCC123 background, it was possible to observe a variable sensitivity among the different mt tRNA mutants, with the LeuA29G substitution exhibiting the higher sensitivity.

**Figure 7:**
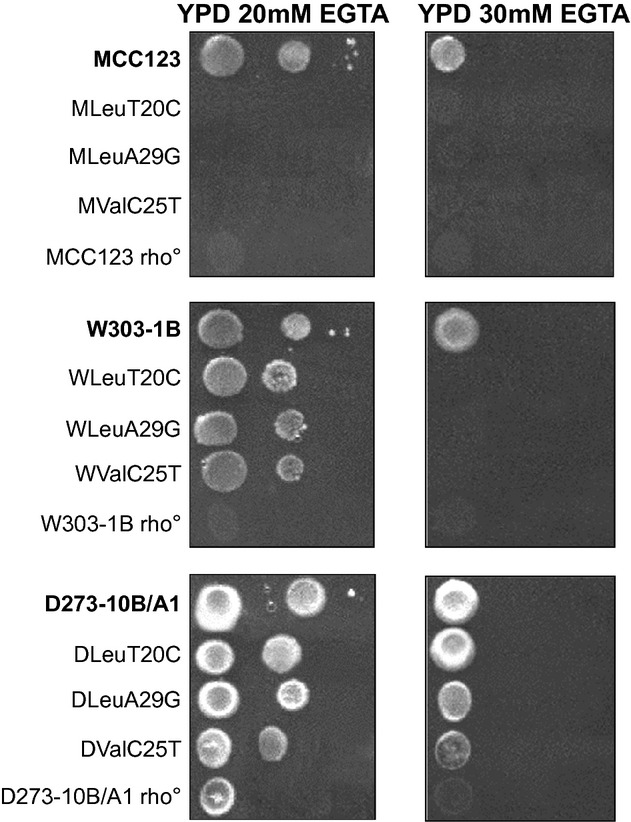
EGTA sensitivity of WT, rho° and mt tRNA mutants in MCC123, W303-1B, and D273-10B/A1 nuclear backgrounds. Exponential cultures grown overnight in YP liquid medium containing 2% glucose, were spotted on plates containing EGTA as indicated. Plates are incubated at 28°C for 4 days.

In addition to respiration, mitochondria also play an important role in regulating calcium homeostasis (Babcock et al. 1997). We investigated whether growth of the WT and mutant strains was equally inhibited by the presence of the cation chelator EGTA. The tests, performed at 28 and 37°C (the latter was not shown because very similar to the former) in YP plates containing 2% glucose, are shown in Fig. 7: WT strains had similar sensitivity to 20 or 30 mM EGTA; on the contrary, tRNA mutants showed a variable sensitivity depending to the nuclear background. The MCC123 mutants were totally sensitive to 20 mM, whereas both the W303-1B and especially to the D273-10B/A1 mutants were resistant to the EGTA. Figure 7 also shows that, within the same nuclear background, the mutated position did not influence the growth capability in the presence of EGTA.

It is interesting to note that by comparing the growth capability of rho° cells, only those isolated from the D273-10B/A1 were resistant to EGTA suggesting that this nuclear background had a very efficient mechanism of intracellular Ca^++^ release also in absence of mt protein synthesis.

### Chronological life span and petite production in WT strains

A number of theories have been proposed to explain the mechanism of aging. According to the theory, proposed by Harman in the 1950s, aging is a consequence of free radical damage. Later, he extended the idea to implicate mt production of ROS in the 1970s. Nowadays, mitochondria functionality is considered a very important contributor to longevity in yeast and in other eukaryotic organisms and is connected with the general problem of diet restriction and aging.

**Figure 8:**
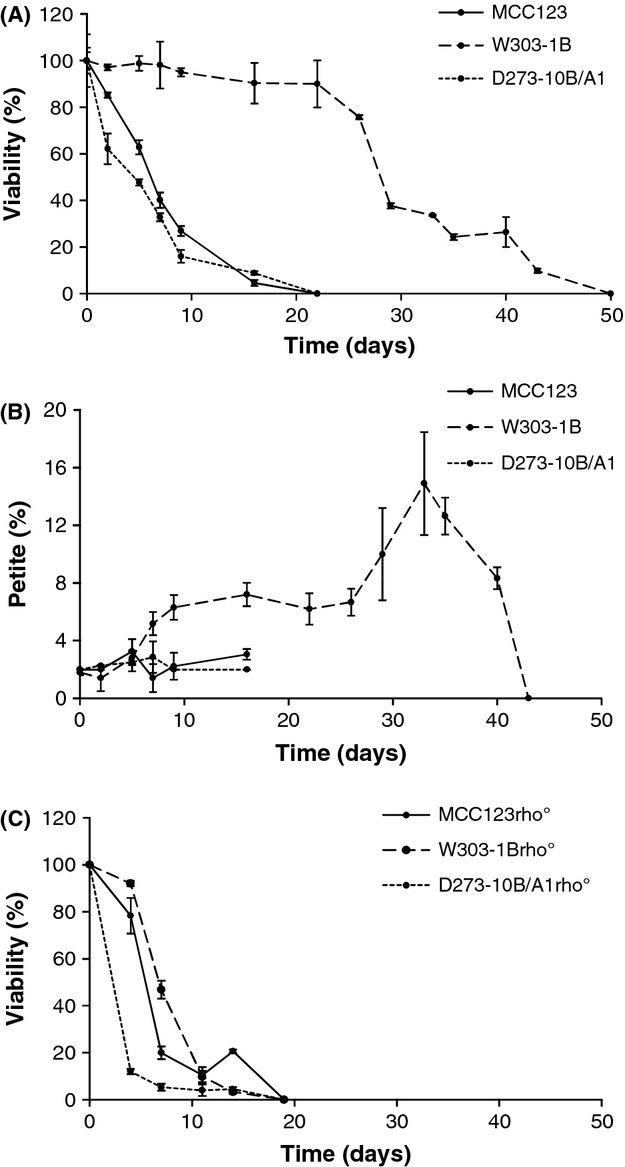
Chronological life span and petite formation. Panel A: comparison of chronological viability of WT strains grown in YP liquid medium containing 2% glucose, reported as percentages of colony forming units. Stationary phases were taken as 100%. Panel B: comparison of petite colonies formation during chronological survival as in panel A. For each analysis (A, B) the same aliquots of cultures were diluted and plated on solid medium. Large and *petite* colonies forming units were counted after 3 days. Panel C: comparison of chronological viability of rho° strains performed as in panel A. Data are means of at least three independent experiments, with reported standard deviations bars.

As a contribution to this open discussion, we wanted to analyze the chronological life span of the three WT strains grown in YP liquid medium containing 2% glucose. Figure 8 (panel A) shows very important differences among the studied strains. In fact, viability of D273-10B/A1 and MCC123 decreased in 15 days, while the viability of W303-1B was almost constant up to 30 days and dropped only after 50 days. Figure 8 (panel B) also shows that in this strain, the production of *petite* colonies increased starting after the fifth day and continued up to the 35th day of incubation. The flat *petite* generation profile of the first 5 days confirmed the similar percentage of *petite* formation (2%) of the three WT strains reported in Table 1.

It might be proposed that *petites* can generate a lower oxidative stress than WT cells (Guidot et al. 1993; Longo et al. 1996) and indeed we noted that the *petite* percentage reached about 7% in the first 26 days of incubation of the W303-1B strain. However, when the W303-1B cells started to die, we observed a further increase in rho^−^/rho° cells (up to 15%) which did not account for the high longevity of W303-1B cells. Interestingly, rho° cells of W303-1B strain showed a much reduced life span, compared to the isogenic WT strain (from 50 to 20 days), whereas the rho° cells of strains D273-10B/A1 and MCC123 maintained a chronological life span similar to their WT (Fig. 8, panel C).

## Discussion

The role of mitochondria in intracellular signaling has implications in development, aging, disease, and environmental adaptation: mt metabolic functions are tightly integrated in cell metabolism and several pathways are known to regulate this integration, which might lead to nuclear control of mt protein synthesis and to retrograde effect of respiratory defects on nuclear genes transcription. Retrograde regulation is broadly defined as cellular responses to changes in the functional state of mitochondria. Due to the multiplicity of organelle functions, a variety of interlinked anterograde and retrograde pathways can be expected (Kelly and Scarpulla 2004). The retrograde response compensates for accumulating mitochondrial dysfunction as yeast cells progress through their replicative life span. This raises the notion that mitochondrial quality control is not sufficient to maintain this organelle over a cell's lifetime. It is consistent with the decline of natural selection along aging and the necessity to devote resources to the survival of young daughter cells and not to the maintenance of old and damaged cells. The link between the retrograde response and the mitochondrial quality control, in the form of autophagy and mitophagy, suggests coordination in the cells. Our study indicates that retrograde regulation might have a relevant importance in the choice of the genetic background for analysis of mt functions. Moreover, differences among various strains might depend on the additive effects of various nuclear genes. At present, researchers have failed to identify a single nuclear gene as major contributor to the variation in the effect of the mt mutations and from our present results no simple and direct relationship can be found.

Variability in regulation of gene expression is also observed in the mt pathologies; the generally accepted explanation for phenotypic diversity of mt DNA mutations is the uneven segregation of mutated and WT mt DNA molecules, with the resulting phenotype being dependent on the ratio of these molecules. However, this explanation is often inadequate for heteroplasmic mutations and inapplicable to homoplasmic mutations. Moreover, homoplasmic mt DNA mutations, showing incomplete penetrance and variability in clinical presentation between patients belonging to the same family, are believed to cause diseases in association with unknown factors (Carelli et al. 2003).

It is known that several laboratory strains are genetically and physiologically heterogeneous, with differences in growth behaviors and chronological life span (Fabrizio and Longo 2007; Ocampo and Barrientos 2011). The S288C derivatives, for example, carry a mutation affecting Hap1, a heme-dependent regulator of a number of genes involved in the aerobic metabolism (Gaisne et al. 1999). This contributes to their reduced ability to respire when compared with strains carrying a wild-type *HAP1* gene, such as W303 (Ocampo et al. 2012). Another variant that may influence mitochondria efficiency has been identified in the sequence of the Tuf1 protein, encoding the mt factor for the mt translation elongation factor, in the D273-10B/A1 strain (Montanari et al. 2013). Moreover, it has been demonstrated that *petite* frequency varies within a large range among laboratory strains (Marmiroli et al. 1980). For example, mt DNA instability of the W303-1B compared to D273-10B/A1 strain has been ascribed to mutations in the *MIP1* gene, which encodes the mt DNA polymerase *γ* (Baruffini et al. 2007).

In summary, we propose the use of the studied yeast strains as follows:

1. W303-1B strain has a very high capability of growth on glycerol medium (Fig. 1) and extended respiration (Fig. 2C) suggesting high efficiency of mitochondrion-related metabolic functions. In the presence of the mt tRNA mutations considered in this study, *petite* production is intermediate (Table 1). This strain can be utilized to study the molecular effect of severe mt tRNA mutations (Montanari et al. 2008) which would have catastrophic effect in other nuclear backgrounds. Kirchman et al. (1999) have demonstrated that retrograde regulation is constitutively active in W303-1B strain and this probably explains why the mt mutations cause a milder phenotype compared with other nuclear backgrounds. An exceptional characteristic of W303-1B strain is the very high chronological life span; it is interesting to note that this strain has also been described to have a high replicative life span, which has been directly correlated to activation of the retrograde regulation (Kirchman et al. 1999). Furthermore, the chronological and the replicative lifespan appear to be regulated by overlapping but distinct mechanisms (Fabrizio and Longo 2003). W303-1B cells are, therefore, the best tool to study the mechanisms and to identify genes involved in longevity; in particular, it will be useful to study which are the changes (at transcriptional and expression levels) that occur after 25 days of incubation.
2. D273-10B/A1 strain has the higher growth rate in glucose-containing medium (Fig. 3A) with a very rapid O_2_ consumption, poorly influenced by glucose concentration (Figs 3B and 4). The analyzed mt tRNA mutations do not increase petite production (Table 1), but have deleterious effect on the capacity of growth in medium containing either 3% glycerol or low glucose concentration (Fig. 2C, D and 4). We might hypothesize that in this strain the retrograde effect is absent or strongly reduced. It can be used to study mutations with mild effect on mt functions.
3. MCC123 strain has the lowest growth rate in glycerol medium (Fig. 1). The studied mt tRNA mutations increase the percentage of petite cells and this value depends on the severity of the mutation (Table 1). Therefore, the MCC123 might be preferentially used to test the phenotypic effects of tRNA substitutions and to study the molecular effect of mutations that cause severe impair of mt protein synthesis. Moreover, MCC123 cells have been used to isolate nuclear suppressor genes that, when overexpressed, rescued the defective growth phenotype of the tRNA mutants (De Luca et al. 2006). In fact, we note that the efficiency of suppression is also strain-dependent as mutants with D273-10B/A1 nuclear background were never rescued by suppressor genes.
4. Stress assays show that the three WT strains have different resistance to EGTA suggesting that probably the regulation of specific pathways is characteristic for each strain. For example: (i) the MCC123 nuclear background is the only one in which the presence of mt tRNA mutations cause a complete sensitivity to EGTA; (ii) the D273-10B/A1 nuclear background causes a partial sensitivity to EGTA only in the complete impairment of mt protein synthesis (rho° cells).

It is well-known that yeast is a very good model to understand the molecular pathways and mechanisms involved in health and diseases. Many genes and regulatory mechanisms are evolutionarily conserved so that the knowledge gained using this simple eukaryote can be extrapolated to metazoans (Johnson et al. 2013). Finally, the genetic variability in yeast strains may offer important opportunities as each of them can be more profitably used to investigate specific pathways.

## References

[b1] Babcock DF, Herrington J, Goodwin PC, Park YB, Hille B (1997). Mitochondrial participation in the intracellular Ca2+ network. J. Cell Biol.

[b2] Baruffini E, Ferrero I, Foury F (2007). Mitochondrial DNA defects in *Saccharomyces cerevisiae* caused by functional interactions between DNA polymerase gamma mutations associated with diseases in human. Biochim. Biophys. Acta.

[b3] Carelli V, Giordano C, d'Amati G (2003). Pathogenic expression of homoplasmic mtDNA mutations needs a complex nuclear-mitochondrial interaction. Trends Genet.

[b4] De Luca C, Besagni C, Frontali L, Bolotin-Fukuhara M, Francisci S (2006). Mutations in yeast mt tRNAs: specific and general suppression by nuclear encoded tRNA interactors. Gene.

[b5] De Luca C, Zhou Y, Montanari A, Morea V, Oliva R, Besagni C (2009). Can yeast be used to study mitochondrial diseases? Biolistic tRNA mutants for the analysis of mechanisms and suppressors. Mitochondrion.

[b6] Doimo M, Trevisson E, Sartori G, Burlina A, Salviati L (2012). Yeast complementation is sufficiently sensitive to detect the residual activity of ASL alleles associated with mild forms of arginosuccinic aciduria. J. Inherit. Metab. Dis.

[b7] Elelj-Fridhi N, Pallier C, Zelikson R, Guetari M, Bolotin-Fukuhara M (1991). Mutational studies of the major tRNA region of the *S. cerevisiae* mitochondrial genome. Curr. Genet.

[b8] Fabrizio P,, Longo VD (2003). The chronological life span of *Saccharomyces cerevisiae*. Aging Cell.

[b9] Fabrizio P,, Longo VD (2007). The chronological life span of *Saccharomyces cerevisiae*. Methods Mol. Biol.

[b10] Facchin S, Lopreiato R, Ruzzene M, Marin O, Sartori G, Götz C (2003). Functional homology between yeast piD261/Bud32 and human PRPK: both phosphorylate p53 and PRPK partially complements piD261/Bud32 deficiency. FEBS Lett.

[b11] Feuermann M, Francisci S, Rinaldi T, De Luca C, Rohou H, Frontali L (2003). The yeast counterparts of human ‘MELAS’ mutations cause mitochondrial dysfunction that can be rescued by overexpression of the mitochondrial translation factor EF-Tu. EMBO Rep.

[b12] Gaisne M, Becam AM, Verdiere J, Herbert C (1999). A ‘natural’ mutation in *Saccharomyces cerevisiae* strains derived from S288c affects the complex regulatory gene HAP1 (CYP1). Curr. Genet.

[b13] Guidot DM, McCord JM, Wright RM, Repine JE (1993). Absence of electron transport (Rho 0 state) restores growth of a manganese-superoxide dismutase-deficient *Saccharomyces cerevisiae* in hyperoxia. Evidence for electron transport as a major source of superoxide generation in vivo. J. Biol. Chem.

[b14] Johnson SC, Rabinovitch PS, Kaeberlein M (2013). mTOR is a key modulator of ageing and age-related disease. Nature.

[b15] Kelly DP,, Scarpulla RC (2004). Transcriptional regulatory circuits controlling mitochondrial biogenesis and function. Genes Dev.

[b16] Kirchman PA, Kim S, Lai C, Jazwinski SM (1999). Interorganelle signaling is a determinant of longevity in *Saccharomyces cerevisiae*. Genetics.

[b17] Koutnikova H, Campuzano V, Foury F, Dollé P, Cazzalini O, Koenig M (1997). Studies of human, mouse and yeast homologues indicate a mitochondrial function for frataxin. Nat. Genet.

[b18] Lindegren CC, Lindegren G (1971). Thallium as a vital stain for yeast mitochondria. Nature.

[b19] Longo VD, Gralla EB, Valentine JS (1996). Superoxide dismutase activity is essential for stationary phase survival in *Saccharomyces cerevisiae*. J. Biol. Chem.

[b20] Marmiroli N, Donnini C, Restivo FM, Tassi F, Puglisi PP (1980). Analysis of rho mutability in *Saccharomyces cerevisiae*. II. Role of the mitochondrial protein synthesis. Mol. Gen. Genet.

[b21] Montanari A, Besagni C, De Luca C, Morea V, Oliva R, Tramontano A (2008). Yeast as a model of human mitochondrial tRNA base substitutions: investigation of the molecular basis of respiratory defects. RNA.

[b22] Montanari A, De Luca C, Frontali L, Francisci S (2010). Aminoacyl-tRNA synthetases are multivalent suppressors of defects due to human equivalent mutations in yeast mt tRNA genes. Biochim. Biophys. Acta.

[b23] Montanari A, De Luca C, Di Micco P, Morea V, Frontali L, Francisci S (2011). Structural and functional role of bases 32 and 33 in the anticodon loop of yeast mitochondrial tRNA Ile. RNA.

[b24] Montanari A, Zhou YF, Fazzi D'Orsi M, Bolotin-Fukuhara M, Frontali L, Francisci S (2013). Analyzing the suppression of respiratory defects in the yeast model of human mitochondrial tRNA diseases. Gene.

[b25] Mortimer RK,, Johnston JR (1986). Genealogy of principal strains of the yeast genetic stock center. Genetics.

[b26] Mulero JJ,, Fox TD (1993). Alteration of the *Saccharomyces cerevisiae* COX2 mRNA 5'-untranslated leader by mitochondrial gene replacement and functional interaction with the translational activator protein PET111. Mol. Biol. Cell.

[b27] Myers AM, Pape LK, Tzagoloff A (1985). Mitochondrial protein synthesis is required for maintenance of intact mitochondria genomes in *S. cerevisiae*. EMBO J.

[b28] Ocampo A,, Barrientos A (2011). Quick and reliable assessment of chronological life span in yeast cell populations by flow cytometry. Mech. Ageing Dev.

[b29] Ocampo A, Liu J, Schroeder EA, Shadel GS, Barrientos A (2012). Mitochondrial respiratory thresholds regulate yeast chronological lifespan and its extension by caloric restriction. Cell Metab.

[b30] Panizza E, Ercolino T, Mori L, Rapizzi E, Castellano M, Opocher G (2013). Yeast model for evaluating the pathogenic significance of SDHB, SDHC and SDHD mutations in PHEO-PGL syndrome. Hum. Mol. Genet.

[b31] Rafael J,, Nicholls DG (1984). Mitochondrial membrane potential monitored in situ within isolated guinea pig brown adipocytes by a styryl pyridinium fluorescent indicator. FEBS Lett.

[b32] Sherman F,, Slonimski PP (1964). Respiration deficient mutants of yeast. II Biochemistry. Biochim. Biophys. Acta.

[b33] Sherman F, Stewart JW, Parker JH, Inhaber E, Shipman NA, Putterman GJ (1968). The mutational alteration of the primary structure of yeast iso-1-cytochrome c. J. Biol. Chem.

[b34] Sprinzl M,, Vassilenko KS (2005). Compilation of tRNA sequences and sequences of tRNA genes. Nucleic Acids Res.

[b35] Thomas BJ, Rothstein R (1989). The genetic control of direct-repeat recombination in *Saccharomyces*: the effect of rad52 and rad1 on mitotic recombination at GAL10, a transcriptionally regulated gene. Genetics.

[b36] Tzagoloff A, Akai A, Needleman RB (1975). Assembly of the mitochondrial membrane system. Characterization of nuclear mutants of *Saccharomyces cerevisiae* with defects in mitochondrial ATPase and respiratory enzymes. J. Biol. Chem.

[b37] Valente L, Tiranti V, Marsano RM, Malfatti E, Fernandez-Vizarra E, Donnini C (2007). Infantile encephalopathy and defective mitochondrial DNA translation in patients with mutations of mitochondrial elongation factors EFG1 and EFTu. Am. J. Hum. Genet.

